# Microarray analysis of gene expression in the diacylglycerol kinase η knockout mouse brain

**DOI:** 10.1016/j.bbrep.2019.100660

**Published:** 2019-06-25

**Authors:** Suguru Komenoi, Yuji Suzuki, Maho Asami, Chiaki Murakami, Fumi Hoshino, Sohei Chiba, Daisuke Takahashi, Sayaka Kado, Fumio Sakane

**Affiliations:** a Department of Chemistry, Graduate School of Science, 1-33 Yayoi-cho, Inage-ku, Chiba, 263-8522, Japan; b Center for Analytical Instrumentation, Chiba University, 1-33 Yayoi-cho, Inage-ku, Chiba, 263-8522, Japan

**Keywords:** Diacylglycerol kinase, Bipolar disorder, Prolactin, Growth hormone, Phosphatidic acid, Lysophosphatidic acid, BPD, bipolar disorder, DAVID, Database for AnnotationVisualization and Integrated Discovery, DG, diacylglycerol, DGK, diacylglycerol kinase, ERK, extracellular signal-regulated kinase, Fpr2, N-formyl peptide receptor 2, Gh, growth hormone, Glp1r, glucagon-like peptide 1 receptor, GO:BP, Gene Ontology: Biological Process, GWAS, genome-wide association study, Il1b, interleukin 1β, KEGG, Kyoto Encyclopedia of Genes and Genomes, KO, knockout, LC-MS, liquid chromatography-mass spectrometry, LPA, lysophosphatidic acid, MEK, mitogen-activated protein kinase/ERK kinase, PA, phosphatidic acid, PI, phosphatidylinositol, Prl, prolactin, PUFA, polyunsaturated fatty acid, SERT, serotonin transporter, WT, wild type

## Abstract

We have revealed that diacylglycerol kinase η (DGKη)-knockout (KO) mice display bipolar disorder (BPD) remedy-sensitive mania-like behaviors. However, the molecular mechanisms causing the mania-like abnormal behaviors remain unclear. In the present study, microarray analysis was performed to determine global changes in gene expression in the DGKη-KO mouse brain. We found that the DGKη-KO brain had 43 differentially expressed genes and the following five affected biological pathways: “neuroactive ligand-receptor interaction”, “transcription by RNA polymerase II”, “cytosolic calcium ion concentration”, “Jak-STAT signaling pathway” and “ERK1/2 cascade”. Interestingly, mRNA levels of prolactin and growth hormone, which are augmented in BPD patients and model animals, were most strongly increased. Notably, all five biological pathways include at least one gene among prolactin, growth hormone, forkhead box P3, glucagon-like peptide 1 receptor and interleukin 1β, which were previously implicated in BPD. Consistent with the microarray data, phosphorylated ERK1/2 levels were decreased in the DGKη-KO brain. Microarray analysis showed that the expression levels of several glycerolipid metabolism-related genes were also changed. Liquid chromatography-mass spectrometry revealed that several polyunsaturated fatty acid (PUFA)-containing phosphatidic acid (PA) molecular species were significantly decreased as a result of DGKη deficiency, suggesting that the decrease affects PUFA metabolism. Intriguingly, the PUFA-containing lysoPA species were markedly decreased in DGKη-KO mouse blood. Taken together, our study provides not only key broad knowledge to gain novel insights into the underlying mechanisms for the mania-like behaviors but also information for developing BPD diagnostics.

## Highlights

• mRNA levels of prolactin and growth hormone were strongly increased in DGKη-KO mice.
• An annotation of the results identified several affected biological pathways.
• Phosphorylated ERK1/2 levels were decreased in the DGKη-KO brain.
• PUFA-containing phosphatidic acid (PA) species were decreased by DGKη-KO.
• PUFA-containing lysoPA species were decreased in DGKη-KO mouse blood.

## Graphical abstract

**Figure:**
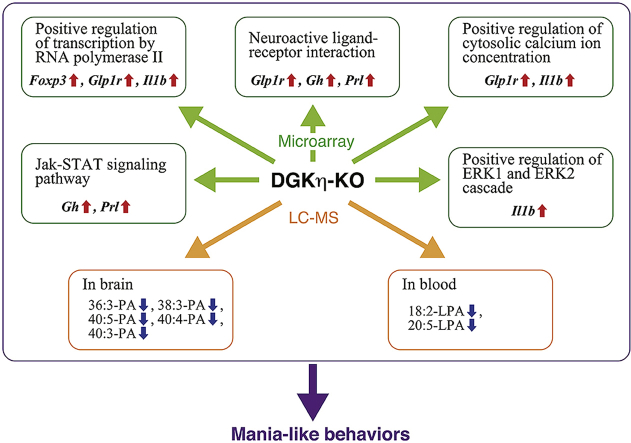

## 1 Introduction

Bipolar disorder (BPD) is a mental disorder characterized by unusual shifts in mood from the heights of mania to the depths of depression [1]. Current reports indicate that the lifetime prevalence of BPD may be closer to 5%. Because of the elevated morbidity and mortality suffered by individuals with BPD, the disorder has been increasingly recognized as a major health problem. Particularly, 25%–50% of patients with BPD attempt suicide at least once over their lifetimes, and 8%–19% will complete suicide [2]. Despite advances in its diagnosis, the underlying neurobiology of BPD remains largely unknown. Therefore, there are only suboptimal treatment options. Moreover, biomarkers for onset and progression, which are essential for precise diagnosis, are presently lacking. Taken together, BPD is still an unmet medical need.

Recently, several genome-wide association studies (GWASs) conducted by Baum et al. [3], Weber et al. [4] and Zeng et al. [5] have consecutively showed that *DGKH* (diacylglycerol kinase (DGK) η gene) is associated with the etiology of BPD. Moreover, *DGKH* is located within the BPD linkage region on 13q14 [6,7]. Furthermore, Moya et al. reported that DGKη mRNA levels were significantly increased in patients with BPD (many of whom are likely in a depressive or normal state because the manic state is often brief) [8]. Therefore, *DGKH* is one of the few replicated risk genes for BPD and is attracting much attention as a BPD-associated gene [9].

DGK phosphorylates diacylglycerol (DG) to produce phosphatidic acid (PA) [[10], [11], [12], [13]]. To date, ten DGK isozymes have been identified [[10], [11], [12], [13]]. The η isozyme of DGK [14,15] belongs to type II DGKs [16], and has a pleckstrin homology domain at its N-terminal and a catalytic domain that is divided into two subdomains. We reported that DGKη is required for the Ras–B-Raf–C-Raf–mitogen-activated protein kinase/ERK kinase (MEK)–extracellular signal-regulated kinase (ERK) signaling cascade in cancer-derived cells [17]. DGKη is most abundantly expressed in the brain [14,18]. Moreover, DGKη was strongly detected in layers II–VI of the cerebral cortex; in the CA1, CA2 and dentate gyrus regions of the hippocampus; in the mitral cells and glomerular layer of the olfactory bulb; and in the Purkinje cells in the cerebellum of one- to 32-week-old mice [18]. We recently found that the pleckstrin homology domain of DGKη is bound to phosphatidylinositol (PI) 4,5-bisphosphate [19], which is an important component of PI turnover [20].

Recently, we generated DGKη-knockout (KO) mice and performed behavioral tests [21]. Intriguingly, DGKη-KO mice displayed an overall behavioral profile that is similar to the manic state of BPD, including hyperactivity, reduced anxiety and lower depressive states. Moreover, these phenotypes were significantly attenuated by the administration of lithium, a therapeutic agent for BPD (mania) [21]. These results strongly implied the existence of a relationship between BPD and DGKη. However, the molecular mechanisms explaining how mania-like behaviors were caused by DGKη deficiency remains unknown.

The purpose of the present work was to elucidate differences between the brains of DGKη-KO mice and wild-type (WT) sibling controls. To determine this, we performed a microarray analysis. The analysis revealed that the deficiency of DGKη appears to modulate gene expression in five biological pathways including “neuroactive ligand-receptor interaction”, “positive regulation of transcription by RNA polymerase II”, “positive regulation of cytosolic calcium ion concentration”, “Jak-STAT signaling pathway” and “Positive regulation of ERK1 and ERK2 cascade”. Notably, all five biological pathways include at least one gene among *Prl* (prolactin), *Gh* (growth hormone), *Foxp3* (forkhead box P3)*, Glp1r* (glucagon-like peptide 1 receptor) and *Il1b* (interleukin 1β), which have previously been implicated in BPD. Regarding glycerolipid metabolism, the expression levels of several glycerolipid-related genes were changed. We found that polyunsaturated fatty acid (PUFA)-containing PA species, such as 18:1/18:2-, 18:0/20:3-, 18:0/22:5-, 20:0/20:4- and 18:1/22:2-PA, were significantly decreased in the DGKη-KO mouse brain and that 18:2- and 20:5-lysoPA (LPA) were markedly decreased in DGKη-KO mouse blood.

## 2 Materials and methods

### 2.1 Mice

This study received approval from the Animal Experiment Committee of Chiba University (permission number: 28–77 and 29–195 and 30–185). All procedures relating to animal care and treatment were conducted in compliance with the National Institutes of Health: Guide for the Care and Use of Laboratory Animals. The mice (male, 12-week-old, ~25 g) were housed in groups of 3–4 in standard cages at 24 ± 2 °C under a 12 h light–dark cycle (lights on from 7:00 to 19:00) with ad libitum access to food and water. DGKη-KO mice (*dgkh*^–^/^–^, Accession No. CDB0606K) were generated as previously described [21]. In brief, we deleted part of the catalytic domain encoded by exons 5 and 6 of the DGKη gene in mice by homologous recombination. We confirmed that DGKη protein was not detectable in DGKη-KO mice [21]. DGKη-KO mice were originated from C57BL/6 and CBA background, and then backcrossed with C57BL/6 mice (Japan SLC, Hamamatsu, Japan). We used WT (C57BL/6) male littermates as a control group for DGKη-KO mice.

### 2.2 Microarray analysis

Cerebral cortexes were homogenized in QIAzol lysis reagent (Qiagen, Hilden, Germany), and total RNA was isolated with the Direct-zol™ RNA Miniprep (ZYMO RESEARCH, Irvine, CA). Microarray hybridization (SurePrint G3 Mouse Gene Expression 8 × 60K Microarray, Agilent, Santa Clara, CA, USA) was performed at the TaKaRa Bio (Kusatsu, Japan). Three experiments using independent cohorts were carried out.

Differential gene expression in DGKη-KO mice was normalized to WT data (WT = 1 (log_2_1 = 0)) to obtain the fold-change values of all genes. The values are presented as the mean (log_2_) ± SEM of three independent experiments. Statistical comparisons were performed using Student's t-tests. A cut-off *P*-value <0.05 and fold-change ≥ absolute 2 (log_2_2 = 1 or log_2_1/2 = −1) were applied.

### 2.3 Analysis of biological processes

Functional annotation lists of Gene Ontology: Biological Process (GO:BP) and Kyoto Encyclopedia of Genes and Genomes (KEGG) categories were generated in the Database for Annotation, Visualization and Integrated Discovery (DAVID: https://david.ncifcrf.gov/).

### 2.4 Reverse transcription polymerase chain reaction (RT-PCR)

Total RNA isolation, reverse transcription and PCR amplification were performed as previously described [22]. PCR amplification was performed using rTaq polymerase (Toyobo, Osaka, Japan) and the following mouse *Prl*-, *Gh*- and glyceraldehyde 3-phosphate dehydrogenase (GAPDH)-specific oligonucleotide primers in the outlined conditions: *Prl*, forward primer (5′-ATGACCATGAACAGCCAGGGGT-3′) and reverse primer (5′-TTCCTCAATCTCTTTGGCTCTTGATAGGAT-3′) with PCR conditions of 94 °C for 3 min, then 30 (DGKη-KO) or 35 (WT) cycles of 94 °C for 30 s, 58 °C for 30 s, and 72 °C for 1 min, and finally 72 °C for 5 min; *Gh*, forward primer (5′-ATGGCTACAGACTCTCGGACCTC-3′) and reverse primer (5′-CTGCATCAGAGCCTGGATGCC-3′) with PCR conditions of 94 °C for 3 min, then 34 (DGKη-KO) or 39 (WT) cycles of 94 °C for 30 s, 53 °C for 30 s, and 72 °C for 1 min, and finally 72 °C for 5 min; GAPDH, forward primer (5′-TCGGTGTGAACGGATTTGGCCGTATT-3′) and reverse primer (5′-CATGTAGGCCATGAGGTCCACCAC-3′) with PCR conditions of 94 °C for 3 min, then 35 cycles of 94 °C for 30 s, 58 °C for 30 s, and 72 °C for 1 min; and finally 72 °C for 5 min. The amplified PCR products were separated by agarose gel electrophoresis and stained with ethidium bromide (Wako Pure Chemical Industries, Osaka, Japan).

### 2.5 Western blotting

Western blotting of brain tissues were performed as previously described [18]. The cerebral cortexes of mice were homogenized in lysis buffer (50 mM HEPES, pH7.2, 150 mM NaCl, 5 mM MgCl_2_) containing cOmplete™ EDTA-free protease inhibitor (Roche Diagnostics, Basel, Switzerland)) and centrifuged at 1,000 *g* for 5 min. The tissue lysates (20 μg of protein) were separated on SDS-PAGE (10% acrylamide gel). The separated proteins were transferred to a polyvinylidene difluoride membrane (Pall Life Sciences, Port Washington, NY). The membrane was blocked with 5% skim milk and incubated with an anti-ERK1/2 antibody (1:1000 dilution, BRID: AB_330744, Cell Signaling Technology, Danvers, MA), an anti-phospho-ERK1/2 antibody (1:1000 dilution, BRID: AB_331646, Cell Signaling Technology) or an anti-β-actin polyclonal antibody (1:1000 dilution, BRID: AB_476693, Sigma-Aldrich, St Louis, MO) overnight at 4 °C. The immunoreactive bands were visualized using a peroxidase-conjugated anti-rabbit IgG antibody (1:10000 dilution, BRID: AB_2337943, Jackson ImmunoResearch Laboratories, West Grove, PA) and the ECL Western Blotting Detection System (GE Healthcare Bio-Sciences, Piscataway, NJ).

### 2.6 Lipid extraction

The cerebral cortexes of mice were homogenized in ice-cold lysis buffer (50 mM HEPES, pH 7.2, 150 mM NaCl, 5 mM MgCl_2_ and cOmplete™ EDTA-free protease inhibitor, followed by centrifugation at 1000×*g* for 5 min at 4 °C. Total lipids were extracted from the mouse brain (cerebral cortex) according to the method of Bligh and Dyer [23]. 2 ml of methanol and 1 ml of chloroform were added to the 700 μl of sample. 100 μl of 3 M HCl was added to the sample in order to improve recovery ratio of acidic phospholipids [24]. The solvent containing lipids was dried under N_2_ gas, and the extracted lipids were reconstituted in 100 μl of chloroform/methanol (2:1, v/v).

### 2.7 Liquid chromatography (LC)-mass spectrometry (MS)

PA species in extracted cellular lipids (10 μl) containing 40 pmol of the 14:0/14:0-PA internal standard (Sigma-Aldrich, St. Louis, MO, USA) were analyzed by LC-MS using an Accela LC system (Thermo Fisher Scientific, Waltham, MA, USA) equipped with a Unison UK-Silica column (3 μm, 150 × 2.0 mm i.d., Imtakt, Kyoto, Japan) and coupled online to an Exactive Orbitrap MS (Thermo Fisher Scientific) equipped with an electrospray ionization source as described previously [24]. A binary gradient consisting of two solvents: solvent A (chloroform/methanol (89:10) containing 0.28% ammonia) and solvent B (chloroform/methanol/water (55:39:5) containing 0.28% ammonia). The gradient elution program was 20% B for 5 min, 20%–30% B for 10 min, 30%–60% B for 25 min, 60%–100% B for 5 min, 100% B for 25 min, followed by 100%–20% B for 1 min. The flow rate was 0.3 ml/min, and chromatography was performed at 25 °C. The MS peaks are presented in the form of X:Y, where X is the total number of carbon atoms and Y is the total number of double bonds in both acyl chains of the PA. All LC-MS data were normalized based on the inorganic phosphate content and the intensity of the internal standard.

### 2.8 Lipid extraction and analysis of LPA molecular species

Total lipids were extracted from mouse serum according to the method of Bligh and Dyer [23]. LPAs in extracted serum lipids (10 μl) containing 4 pmol of the 17:0-LPA internal standard (Sigma-Aldrich) were analyzed with the LC-MS described above.

### 2.9 LC-MS/MS

For the identification of fatty acid residues in PA molecular species by LC-MS/MS. Total lipids were extracted from the cerebral cortexes as described above. The extracted lipids (10 μl) were separated on a LC system (Shimadzu Corporation, Kyoto, Japan) using a Unison UK-Silica column (3 μm, 150 × 2.0 mm i.d., Imtakt, Kyoto, Japan) as described previously [24]. This LC system was controlled by the Analyst^®^ software (AB SCIEX, Washington D.C., USA). A binary gradient consisting of two solvents: solvent A (chloroform/methanol (89:10) containing 0.28% ammonia) and solvent B (chloroform/methanol/water (55:39:5) containing 0.28% ammonia). The gradient elution program was 20% B for 5 min, 20%–30% B for 10 min, 30%–60% B for 25 min, 60% for 5 min, 60%–20% B for 1 min, followed by 20% B for 14 min. The flow rate was 0.3 ml/min, and chromatography was performed at 25 °C.).

The LC system was coupled online to Triple Quad™ 4500 (AB SCIEX), a triple-quadrupole tandem mass spectrometer equipped with turbo spray ionization source. The experimental conditions are: ion spray voltage −4500 v, curtain gas 30 psi, collision gas 6 psi, temperature 300 °C, declustering potential −160 v, entrance potential −10 v, collision energy −42 v, collision cell exit potential −11 v, ion source gas I 70 psi and ion source gas II 30 psi. PA molecular species were detected in a multiple reaction monitoring (MRM) mode.

### 2.10 Statistical analysis

The Kolmogorov-Smirnov test was carried out to assess the normality of data. Statistical comparisons were performed using a two-tailed *t*-test or one-way ANOVA followed by Tukey's *post hoc* test (Prism 5, GraphPad Software, La Jolla, CA, USA). No sample size calculation was performed. No test for outliners was conducted.

## 3 Results

### 3.1 Differential gene expression in DGKη-KO mice

To determine differences in gene expression between the cerebral cortexes of DGKη-KO male mice and those of WT sibling controls (three animals per genotype (WT: n = 3, KO: n = 3)), we performed a microarray analysis. To obtain a broad overview of differential gene expression, all genes meeting the cut-off values (*P*-value <0.05 and fold-change ≥ absolute 2 (log_2_ 1 or log_2_ –1)) in DGKη-KO mice were analyzed. There was a total of 43 (26 up- and 17 downregulated) differently regulated genes in DGKη-KO mice (Table 1). Overall, there were considerably more upregulated genes than downregulated genes.

**Table 1:** Changes in gene expression in DGKη-KO mouse cerebral cortex.

Gene	Gene name	Fold-change (log_2_)	SEM	P value
*Prl*	prolactin	5.4	±1.7	0.031
*Gh*	growth hormone	5.3	±1.8	0.042
*Adamts19*	a disintegrin-like and metallopeptidase (reprolysin type) with thrombospondin type 1 motif, 19	2.9	±0.4	0.002
*Kqt2*	potassium channel, KvQLT family	2.3	±0.7	0.032
*Foxp3*	forkhead box P3	2.3	±0.7	0.031
*AMPD3*	adenosine monophosphate deaminase 3	2.2	±0.3	0.003
*Top2a*	topoisomerase (DNA) II alpha	1.5	±0.4	0.026
*Smim6*	small integral membrane protein 6	1.5	±0.2	0.002
*Tomt*	transmembrane O-methyltransferase	1.5	±0.3	0.007
*Eqtn*	equatorin, sperm acrosome associated	1.5	±0.5	0.037
*Cnot4*	CCR4-NOT transcription complex subunit 4	1.4	±0.1	0.001
*Fpr2*	N-formyl peptide receptor 2	1.4	±0.2	0.001
*Glp1r*	glucagon-like peptide 1 receptor	1.4	±0.4	0.022
*Hif3a*	hypoxia inducible factor 3, alpha subunit	1.2	±0.1	<0.001
*Atp13a4*	ATPase type 13A4	1.2	±0.0	<0.001
*Arg1*	arginase	1.2	±0.2	0.004
*Slc5a8*	solute carrier family 5 (iodide transporter), member 8	1.2	±0.4	0.040
*Lrrc17*	leucine rich repeat containing 17	1.2	±0.3	0.009
*Bub1*	BUB1, mitotic checkpoint serine/threonine kinase	1.2	±0.4	0.037
*Ppp1r3e*	protein phosphatase 1, regulatory (inhibitor) subunit 3E	1.2	±0.3	0.017
*Spaca1*	sperm acrosome associated 1	1.1	±0.2	0.003
*Il1b*	interleukin 1 beta	1.1	±0.2	0.011
*Rhbdl2*	rhomboid like 2	1.1	±0.2	0.012
*Rbmy*	RNA binding motif protein, Y chromosome	1.0	±0.2	0.013
*Dscaml1*	DS cell adhesion molecule like 1	1.0	±0.2	0.010
*Thpo*	thrombopoietin	1.0	±0.3	0.028
*Fut1*	fucosyltransferase 1	−1.0	±0.1	0.003
*Prrg1*	proline rich Gla (G-carboxyglutamic acid) 1	−1.0	±0.2	0.006
*Ces2c*	carboxylesterase 2C	−1.0	±0.2	0.011
*Igk-V21-4*	immunoglobulin kappa chain variable 21 - 4	−1.0	±0.3	0.025
*Gnat3*	guanine nucleotide binding protein, alpha transducing 3	−1.0	±0.2	0.004
*Cryba1*	crystallin, beta A1	−1.0	±0.2	0.002
*Neurog1*	neurogenin 1	−1.0	±0.1	<0.001
*Rnf130*	ring finger protein 130	−1.1	±0.3	0.024
*Defb26*	defensin beta 26	−1.1	±0.2	0.005
*F7*	coagulation factor VII	−1.1	±0.2	0.009
*Siglecf*	sialic acid binding Ig-like lectin F	−1.1	±0.2	0.009
*–*	BApecB1a-P19 IgG heavy chain	−1.2	±0.3	0.016
*Trim63*	tripartite motif-containing 63	−1.2	±0.3	0.014
*Serpinf2*	serine peptidase inhibitor F member 2	−1.2	±0.3	0.015
*Bsx*	brain specific homeobox	−1.2	±0.3	0.009
*Vsig4*	V-set and immunoglobulin domain containing 4	−1.3	±0.4	0.020
*Olr1*	oxidized low density lipoprotein (lectin-like) receptor 1	−1.6	±0.2	0.002

All genes meeting the cut-off (see Materials and Methods) in the DGKη-KO mouse cerebral cortexes were analyzed. The values (fold-change, log_2_) are presented as the mean ± SEM of three animals per genotype (WT: n = 3, KO: n = 3).

To validate the microarray findings, we quantified the expression of two particular genes, *Prl* (prolactin) and *Gh* (growth hormone), which were the most upregulated (log_2_ 5.4 ± 1.7 (absolute 42.2 ± 3.2) fold, *P* = 0.031 and log_2_ 5.3 ± 1.8 (absolute 39.4 ± 3.5) fold, *P* = 0.042, respectively) (Table 1), using RT-PCR. RT-PCR confirmed a similar pattern of upregulated fold-change expression of *Prl* and *Gh* (log_2_ 5.4 ± 1.5 (absolute 42.2 ± 2.8) fold, *P* = 0.004 and log_2_ 4.7 ± 0.8 (absolute 26.0 ± 1.7) fold, *P* = 0.001, respectively) (Suppl. Fig. 1).

**Fig. 1:**
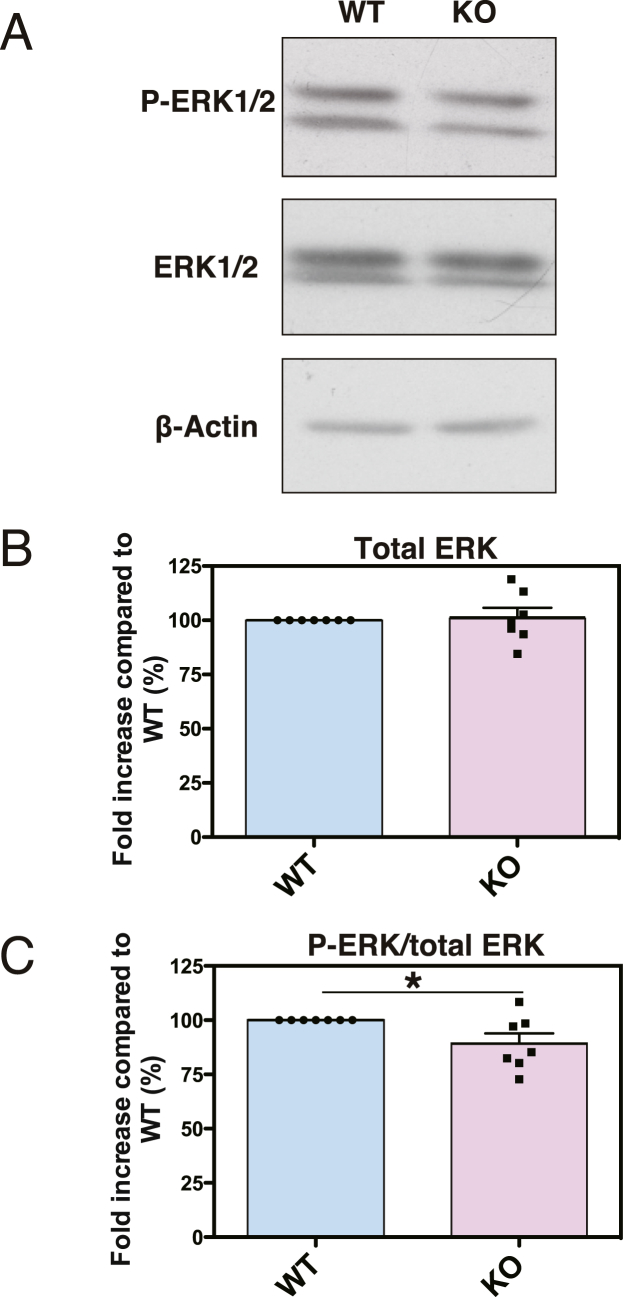
Western blot analysis of phosphorylated ERK1/2. (A) Western blotting of lysates (20 μg) from WT or KO mouse cerebral cortexes was performed to detect total ERK1/2 and phosphorylated ERK1/2 (P-ERK1/2). (B, C) Quantitative analysis of Western blotting (total ERK (B) and P-ERK/total ERK (C)). The bands were scanned and quantified using the ImageJ software. The values of WT mice were set to 1. The values are presented as the mean ± SEM of seven animals per genotype (WT: n = 7, KO: n = 7). *p < 0.05 vs. WT mice.

We analyzed the relationship between BPD and individual genes affected in DGKη-deficient mice (Table 1). *Prl* [25], *Gh* [26], *Il1b* (interleukin 1β) [27], *Foxp3* (forkhead box P3), *Glp1r* (glucagon-like peptide 1 receptor) [28,29] and *Arg1* (arginase) [30], which were intensively increased by DGKη-KO (Table 1), were reported to be related to BPD (see Discussion).

### 3.2 Identification of affected biological processes in DGKη-KO mice

To gain some insight into the biological role of the affected genes found (Table 1) and how they may contribute to BPD remedy (lithium)-sensitive mania-like behaviors caused by DGKη deficiency, we annotated the 43 genes (Table 1) using functional lists of Gene Ontology: Biological Process (GO:BP) and Kyoto Encyclopedia of Genes and Genomes (KEGG) in the DAVID bioinformatics resources. The analysis revealed that the deficiency of DGKη appears to affect gene expression in five biological pathways (Table 2). In addition to the central nerve system-related pathway “neuroactive ligand-receptor interaction (KEGG:mmu04080)”, four pathways such as “positive regulation of transcription by RNA polymerase II (GO:0045944)”, “Jak-STAT signaling pathway (KEGG: mmu04630)”, “positive regulation of cytosolic calcium ion concentration (GO:0007204)” and “positive regulation of ERK1 and ERK2 cascade (GO:0070374)” were affected by DGKη-KO (Table 2).

**Table 2:** Biological pathways affected in the DGKη-KO mouse cerebral cortex.

Database category	Biological pathway	P-value	Gene Count	Genes
GOTERM_BP	Positive regulation of transcription by RNA polymerase II (GO:0045944)	0.004	8	*Bsx*, ***Foxp3***^d^, ***Glp1r***^e^,*Hif3a*, ***Il1b***^f^, *Neurog1*, *Serpinf2*,
KEGG_PATHWAY	Neuroactive ligand-receptor Interaction (mmu04080)	0.031	4	*Fpr2*, ***Glp1r***^e^, ***Gh***^g^,***Prl***^h^
GOTERM_BP	**Positive regulation of cytosolic calcium ion concentration**^a^ (GO:0007204)	0.039	3	*Fpr2*, ***Glp1r***^e^, ***Il1b***^f^,
KEGG_PATHWAY	**Jak-STAT signaling pathway**^b^ (mmu04630)	0.049	3	***Gh***^g^, ***Prl***^h^, *Thpo*
GOTERM_BP	**Positive regulation of ERK1 and ERK2 cascade**^c^ (GO:0070374)	0.059	3	***Il1b***^f^, *Serpinf2*, *Thpo*

For each term, an EASE score cut-off of P-value <0.1 was applied along with gene count ≥3. Along with database category for GO and KEGG using DAVID, Benjamini false discovery rate-corrected P-value, gene count and gene lists are presented. Genes and biological pathways implicated in BPD are indicated in bold font.

a [31].

b [[32], [33], [34]].

c [35,36].

d [37,38].

e [28,29].

f [27].

g [26].

h [39,40].

### 3.3 BPD-related biological processes affected in DGKη-KO mice

We next analyzed the relationship between BPD and biological pathways affected in DGKη-KO mice. As mentioned above, all five biological pathways include at least one gene previously implicated in BPD (Table 2). “Neuroactive ligand-receptor interaction”, which can be directly related to BPD (mania)-like behaviors of DGKη-deficient mice, contains three genes, *Glp1r*, *Gh* and *Prl*, previously implicated with BPD.

In addition to “Neuroactive ligand-receptor interaction”, “Jak-STAT signaling pathway” is influenced by DGKη-KO. This pathway contains *Gh* and *Prl* (Table 2). Intriguingly, the antidepressant actions of current treatments have been shown to be mediated by JAK/STAT-dependent mechanisms [34].

“Positive regulation of cytosolic calcium ion concentration” and “Positive regulation of transcription by RNA polymerase II” are altered by DGKη deficiency (Table 2). It was reported that increased levels of intracellular calcium enhances glycogen synthase kinase (GSK) 3β activity [36], which is closely related to BPD [[41], [42], [43]]. Mitochondrial dysfunctions play a pathophysiological role in some BPD patients by affecting intracellular calcium regulation [31]. A wide variety of impairments in transcription regulation are suggested to be critical to the pathogenesis of BPD. Indeed, this pathway contains three BPD-related genes including *Foxp3*, *Gh* and *Prl* (Table 2).

“Positive regulation of ERK1 and ERK2 cascade” is also affected by DGKη deficiency (Table 2). ERK1/2 was reported to be activated by lithium and valproate, BPD (mania) remedies [35]. The Raf–MEK–ERK1/2 cascade phosphorylates and consequently inhibits GSK3β [36]. Interestingly, we previously reported that DGKη activates the Ras–C-Raf–MEK–ERK1/2 signaling cascade [17] and that DGKη deficiency enhanced GSK3β activity [21].

### 3.4 Effects of DGKη-KO on the ERK1 and ERK2 cascade

Because “Positive regulation of ERK1 and ERK2 cascade” is affected by DGKη deficiency (Table 2) and because DGKη activates the Ras–Raf–MEK–ERK1/2 signaling cascade [17], we examined phosphorylation (activation) levels of ERK1/2 in the DGKη-deficient brains. Indeed, the phosphorylation levels of ERK1/2 were significantly decreased in DGKη-KO mouse cerebral cortexes (89.2 ± 9.8%, P = 0.041) (Fig. 1). Therefore, we confirmed that the ERK1/2 cascade is regulated by DGKη *in vivo*. It is possible that changes in ERK phosphorylation levels in DGKη-KO mice were small (Fig. 1) because DGKη is expressed only in a part of the mouse cerebral cortex [18].

### 3.5 Analysis of PA molecular species in DGKη-KO mouse cerebral cortex

*Olr1* (oxidized low-density lipoprotein (lectin-like) receptor 1) and *Ces2c* (carboxylesterase 2C) (Table 1) are glycerolipid-related genes. *Glp1r* (Table 1, Table 2) is intimately linked to lipid metabolism [44]. Moreover, we noted that the FPR2 (N-formyl peptide receptor 2) [45] (Table 1), which is listed under “Neuroactive ligand-receptor interaction” and “Positive regulation of cytosolic calcium ion concentration” (Table 2), binds to lipoxin A4 derived from PUFA (arachidonic acid). In addition, PUFA is implicated in the pathophysiology and etiology of recurrent mood disorders including BPD [46,47]. Furthermore, DGKη itself is a glycerolipid-metabolizing enzyme that phosphorylates DG to generate PA [[10], [11], [12], [13]]. Therefore, we next investigated the effects of DGKη deficiency on the amounts of PA molecular species, which are the reaction products of DGK.

**Fig. 2:**
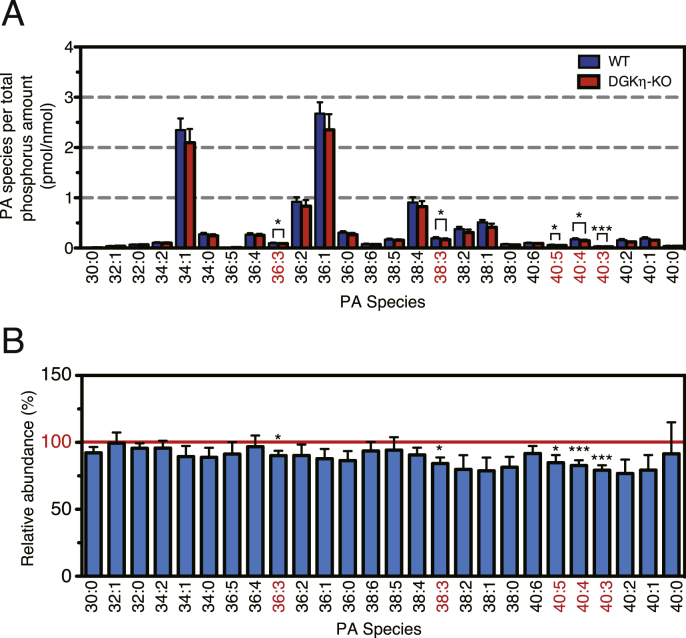
Analysis of PA molecular species in DGKη-KO mouse cerebral cortex. (A) A representative LC-MS analysis result of major PA species from control and DGKη-KO mouse cerebral cortexes. (B) The results are presented as the percentage of the value of each PA molecular species in control mouse cerebral cortex. The values are presented as the mean ± SEM of four animals per genotype (WT: n = 4, KO: n = 4). *p < 0.05, ***p < 0.005 versus control. PA molecular species that were decreased in the DGKη-KO mouse cerebral cortex are indicated with a red font. . (For interpretation of the references to colour in this figure legend, the reader is referred to the Web version of this article.)

We examined whether the amounts of PA molecular species were decreased in the cerebral cortex of the DGKη-KO mouse using a recently established LC-MS method [24,48]. As shown in Fig. 2A, several PA species were reduced. To facilitate comparison, the averages of the relative values (DGKη-KO mice versus control mice) from four independent experiments are displayed (Fig. 2B). In the DGKη-KO mouse cerebral cortexes, several PUFA-containing PA species, such as 36:3 -, 38:3-, 40:5-, 40:4- and 40:3-PA, were significantly decreased (Fig. 2). LC-MS/MS analysis showed that the main molecular species of these PA species were 18:1/18:2 (36:3)-, 18:0/20:3 (38:3)-, 18:0/22:5 (40:5)-, 20:0/20:4 (40:4)- and 18:1/22:2 (40:3)-PA (Suppl. Table 1). These results support our microarray data showing that the deficiency of DGKη affected PUFA-containing glycerophospholipid metabolism. It is likely that changes in the levels of PUFA-containing PA species with KO were relatively small (Fig. 2) because DGKη is expressed only in a part of the mouse cerebral cortex [18] as mentioned above. Moreover, PA species that are generated from multiple pathways, such as the *de novo* synthesis, the hydrolysis of phosphatidylcholine by phospholipase D and the phosphorylation of DG by other DGK isozymes probably caused high background.

### 3.6 Analysis of LPA molecular species in DGKη-KO mouse serum

**Fig. 3:**
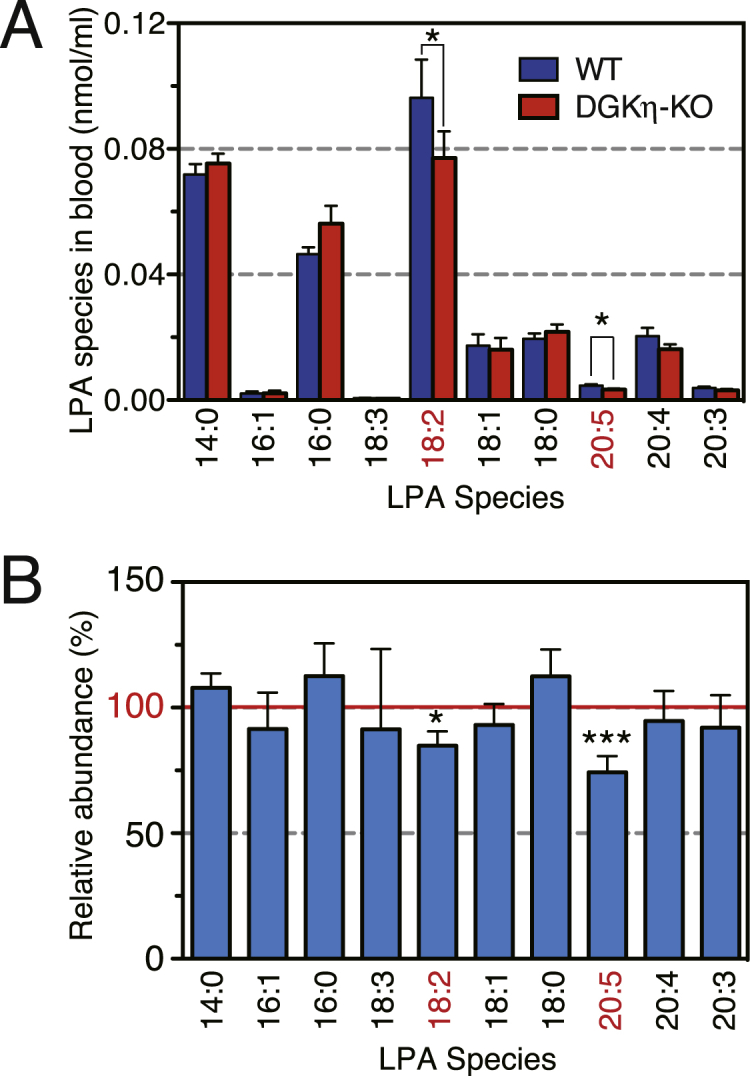
Analysis of LPA molecular species in DGKη-KO mouse serum. (A) A representative LC-MS analysis result of LPA species from control and DGKη-KO mouse serum. (B) The results are presented as the percentage of the value of each LPA molecular species in control mouse serum. The values are presented as the mean ± SEM of six animals per genotype (WT: n = 6, KO: n = 6). *p < 0.05, ***p < 0.005 versus control. LPA molecular species decreased in the DGKη-KO mouse serum are indicated with a red font. . (For interpretation of the references to colour in this figure legend, the reader is referred to the Web version of this article.)

We next examined whether the amounts of LPA species were decreased in DGKη-KO mouse serum using the LC-MS analysis [24,48]. As shown in Fig. 3A, the levels of several LPA species were reduced. To facilitate comparison, the averages of the relative values (DGKη-KO mice versus control mice) from six independent experiments are displayed (Fig. 3B). We found that the amounts of PUFA-containing LPA species 18:2- and 20:5-LPA were significantly decreased in DGKη-KO mouse blood (Fig. 3).

## 4 Discussion

It was strongly suggested that DGKη is one of the key enzymes related to the etiology of BPD. However, the molecular mechanisms by which the DGKη-KO induces mania-like behaviors has been unclear. In the present study, we revealed that the expression levels of 43 genes were changed in the DGKη-KO mouse cerebral cortex (Table 1). Moreover, the database for annotation identified five affected biological pathways. The central nerve system-related pathway “neuroactive ligand-receptor interaction” was annotated (Table 2). Impairments of “Neuroactive ligand-receptor interaction” can be a direct cause of BPD (mania)-like behaviors of DGKη-KO mice.

“Jak-STAT signaling pathway” is affected in DGKη-KO mice (Table 2). Notably, the antidepressant actions of current treatments have been shown to be mediated by JAK/STAT-dependent mechanisms [34]. “Jak-STAT signaling pathway” is implicated in the pathogenesis of inflammatory and autoimmune diseases including rheumatoid arthritis, psoriasis, and inflammatory bowel disease [49]. Il1b (interleukin 1β) and Foxp3 (forkhead box P3) (Table 1, Table 2) are also involved in the immune-inflammatory response system [37,38]. It is interesting to note that there are now several meta-analyses showing that BPD is accompanied by an immune-inflammatory response with increased levels of pro-inflammatory cytokines, acute phase proteins, and other compounds released by an activated immune-inflammatory response system [[32], [33], [34]]. For example, there was a higher incidence in the inflammatory bowel disease cohort compared to controls for BPD [32].

“Positive regulation of ERK1 and ERK2 cascade” is also affected by DGKη deficiency (Table 2). Indeed, we demonstrated that phosphorylation (activation) levels of ERK1/2 were decreased in DGKη-KO mouse cerebral cortexes (Fig. 1). It was reported that ERK was activated by lithium and valproate, BPD (mania) remedies [35]. The B-Raf/C-Raf–MEK–ERK1/2 cascade inhibits GSK3β [36], which is associated with BPD [[41], [42], [43]]. Moreover, B-Raf mRNA and protein levels were significantly reduced in BPD patient brains [50]. It was reported that B-Raf inhibitors stimulate inflammasome activation and IL-1β production [51]. Consistently, DGKη-KO induced IL-1β production (Table 1). Interestingly, we recently found that DGKη enhanced the Ras–B-Raf/C-Raf–MEK–ERK1/2 signaling [17] and that deficiency of DGKη activated GSK3β [21].

Prl (prolactin), which is an anterior pituitary hormone, was intensively increased by DGKη-KO ((log_2_ 5.4 ± 1.7 (absolute 42.2 ± 3.2) fold, Table 1). Prolactin is commonly included in the annotation groups “neuroactive ligand-receptor interaction”, and “Jak-STAT signaling pathway” (Table 2). Prolactin levels were reported to be higher in BPD patients compared to controls [25]. BPD patients undergoing lithium treatment exhibited decreased prolactin levels [25,52]. Moreover, the administration of lithium significantly inhibited prolactin secretion in rats [53]. Intriguingly, lithium attenuated DGKη-deficient-induced mania-like phenotypes as well [21]. In contrast to lithium, it is widely known that many antipsychotic medications were reported to strongly increase prolactin levels in BPD patients in addition to schizophrenia patients [40,54]. The increased prolactin leads to serious side effects. It will be interesting to determine the relationship between the effects of antipsychotic medications and DGKη.

Gh (growth hormone), which is an anterior pituitary hormone, similarly to prolactin, was also strongly increased by DGKη deficiency (log_2_ 5.3 ± 1.8 (absolute 39.4 ± 3.5) fold, Table 1) and is listed under “neuroactive ligand-receptor interaction” and “Jak-STAT signaling pathway” (Table 2). The administration of lithium significantly attenuated GH secretion in addition to prolactin in rats [53]. GH was more strongly increased in manic patients than healthy controls in response to a challenge with a GABA_B_ receptor agonist [26]. There may be similarities between the effects of GABA_B_ receptor agonists for mania and DGKη-KO.

Il1b (interleukin 1β), which was significantly increased in DGKη-KO mouse brains (Table 1), is commonly included in the annotation groups “positive regulation of transcription by RNA polymerase II”, “positive regulation of cytosolic calcium ion concentration”, and “Positive regulation of ERK1 and ERK2 cascade” (Table 2). Il1b, which is a neuroinflammatory marker, was previously reported to increase in the cerebral cortex of BPD patients [27].

Foxp3 (forkhead box P3), which was significantly increased in DGKη-KO mouse brains (Table 1), is listed under “positive regulation of transcription by RNA polymerase II” (Table 2). Foxp3 functions as a master regulator of the regulatory pathway in the development and function of regulatory T cells [55]. It was found that BPD patients exhibited reduced proportions of natural T regulatory cells (CD4^+^ CD25^+^ FoxP3^+^) [37,38].

The expression of Glp1r (glucagon-like peptide 1 receptor), which is annotated in “positive regulation of transcription by RNA polymerase II”, “positive regulation of cytosolic calcium ion concentration” and “neuroactive ligand-receptor interaction” (Table 2), was significantly enhanced (Table 1). Glp1r is widely expressed in the central nervous system. Interestingly, GLP1R agonists were reported to be promising therapeutic options for BPD [28,29].

Arg1 (arginase) was substantially increased in DGKη-KO mouse brains (Table 1). Plasma arginase activities were found to be significantly lower in patients with BPD compared with controls [30].

There are several BPD-related proteins including GSK3β and Clock. Contrary to DGKη, transgenic mice of GSK3β exhibited mania-like behaviors [56,57]. As expected, Prl and Gh levels were significantly lower in rats treated either with AZ1080, a GSK3β-selective inhibitor, or lithium, which also inhibits GSK3β [58], in contrast to DGKη-KO. Mice deficient in Clock also constitute a representative BPD (mania) model [59]. Consistent with DGKη-KO mice, Gh mRNA was increased in the Clock-mutated mouse brains [60].

We found that the deficiency of DGKη decreased PUFA-containing PA species, 36:3 (18:1/18:2)-, 38:3 (18:0/20:3)-, 40:5 (18:0/22:5)-, 40:4 (18:0/22:4)- and 40:3 (18:0/22:3)-PA, in the mouse cerebral cortex (Fig. 2 and Suppl. Table 1). Thus, these PA species may directly affect the functions of BPD-related proteins. Intriguingly, PUFA is implicated in the pathogenesis of BPD [46,47] and our microarray data showed that the gene encoding FPR2 [45], which binds to ligands derived from PUFA, was increased (Table 1). Additionally, GWAS has recently revealed that fatty acid desaturase, which regulates unsaturation of fatty acids, is associated with BPD [61]. These results suggest that DGKη-KO might cause BPD-like behaviors through abnormal PUFA-related glycerophospholipid metabolism affected by decreases in PUFA-containing PA molecular species.

To date, ten DGK isozymes have been identified [[10], [11], [12], [13]]. We previously demonstrated that DGKζ generated 32:0 (16:0/16:0)-PA during Neuro-2a neuroblastoma cell differentiation [62] and that DGKδ produced relatively broad PA species such as 30:0 (14:0/16:0)-, 30:1 (14:0/16:1)-, 32:0 (16:0/16:0)-, 32:1 (16:0/16:1)-, 34:0 (16:0/18:0)- and 34:1 (16:0/18:1)-PA in glucose-stimulated C2C12 myoblasts [48]. These profiles are clearly different from that (36:3 (18:1/18:2)-, 38:3 (18:0/20:3)-, 40:5 (18:0/22:5)-, 40:4 (18:0/22:4)- and 40:3 (18:0/22:3)-PA) of DGKη in the brain (Fig. 2 and Suppl. Table 1). These results further support the fact that DGK isozymes utilize distinct DG molecular species as substrates in different cells under different conditions (stimuli). However, DGKη does not have a preference for different DG species in vitro [63]. Therefore, it is speculated that the enzyme preferentially accesses to a PUFA-containing DG species-rich pool *in vivo*. Alternatively, there could also be changes in the membrane environment that affects the specificity of DGKη as has recently been found with DGKε [64]. DG species derived from PI turnover mainly consist of 18:0/20:4-DG [20]. Therefore, although the pleckstrin homology domain of DGKη interacts with PI4,5-bisphosphate [19], DGKη would utilize DG species supplied from a PI turnover-independent pathway.

LC-MS analysis revealed that the PUFA-containing LPA species, 18:2- and 20:5-LPA, were significantly decreased in DGKη-KO mouse serum (Fig. 3). Particularly, 18:2-LPA was dominantly affected (Fig. 3). The decrease in amounts of PUFA-containing LPA species (18:2-LPA) in DGKη-KO mice (Fig. 3) may be due to the reduction of 18:2-containing PA species such as 18:1/18:2 (36:3)-PA in the brain (Fig. 2 and Suppl. Table 1). In this case, phospholipase A may produce the LPA species via hydrolysis of 18:1/18:2-PA. Alternatively, we revealed that DGKη exhibits 2-monoacylglycerol kinase activity in addition to DGK activity [65], suggesting that the LPA species (18:2-LPA) was generated from 2-monoacylglycerol (18:2-monoacylglycerol) through phosphorylation by DGKη. Similarly, 20:5-LPA (Fig. 3) may be derive from 20:0/20:5 (40:5)-PA (Fig. 2 and Suppl. Table 1) and/or 20:5-monoacylglycerol. Because the DGKη gene was not brain-specifically deleted, we cannot rule out general effects across tissues other than the brain. Notably, the amounts of LPA species in blood are noninvasively measurable. Moreover, DGKη expression is changed in BPD patients [8] and GWASs of BPD have suggested that single nucleotide polymorphisms in the DGKη gene are associated with BPD [[3], [4], [5]]. Therefore, there is the possibility that these LPA species could be novel and useful biomarkers for BPD.

Taken together, our study provides not only key broad knowledge to gain novel insights into the underlying mechanisms of how BPD-related mania-like behaviors were caused by DGKη-KO but also information for developing BPD diagnostics. However, the molecular mechanisms by which DGKη-KO induces mania-like behaviors are not yet fully understood. Further studies will be necessary to explore this issue in more detail.

## Funding

This work was supported in part by grants from MEXT/JSPS KAKENHI Grant Numbers 26291017 (FS), 15K14470 (FS), 17H03650 (FS), and 16J06865 (SKo); the Futaba Electronic Memorial Foundation (FS); the Ono Medical Research Foundation (FS); the Japan Foundation for Applied Enzymology (FS); the Food Science Institute Foundation (FS); the Skylark Food Science Institute (FS); the Asahi Group Foundation (FS); the Japan Milk Academic Alliance (FS); the Japan Food Chemical Research Foundation (FS) and the SENSHIN Medical Research Foundation (FS).

## Author contributions

S.Ko. designed and conducted the experiments, analyzed the data and wrote the manuscript. Y.S., C.M., F.H., M.A., S.C. and S.Ka. performed the experiments and analyzed the data. D.T. designed the study and revised it critically for important intellectual content. F.S. conceived the study and wrote the manuscript.

## Conflicts of interest

The authors declare that they have no conflicts of interest with the contents of this article.

## Appendix A Supplementary data

Supporting Information-190617_V2 Supporting Information-190617_V2

Data Profile.xml Data Profile.xml
