# Supplementary material for: Microarray analysis of gene expression in the diacylglycerol kinase η knockout mouse brain
Source: Biochem Biophys Rep. 2019 Jun 25;19:100660. doi: 10.1016/j.bbrep.2019.100660 (PMC6597918; doi:10.1016/j.bbrep.2019.100660)
Supplement: Supporting Information-190617_V2 [file mmc1.docx]

**Supplemental Materials**

**Microarray analysis of gene expression in the diacylglycerol kinase η knockout mouse brain**

**Suguru Komenoi^1^, Yuji Suzuki^1^, Maho Asami^1^, Chiaki Murakami^1^, Fumi Hoshino^1^, Sohei Chiba^1^, Daisuke Takahashi^1^, Sayaka Kado^2^ and Fumio Sakane^1^***

**^1^** Department of Chemistry, Graduate School of Science, and **^2^** Center for Analytical Instrumentation, Chiba University, 1-33 Yayoi-cho, Inage-ku, Chiba 263-8522, Japan

**
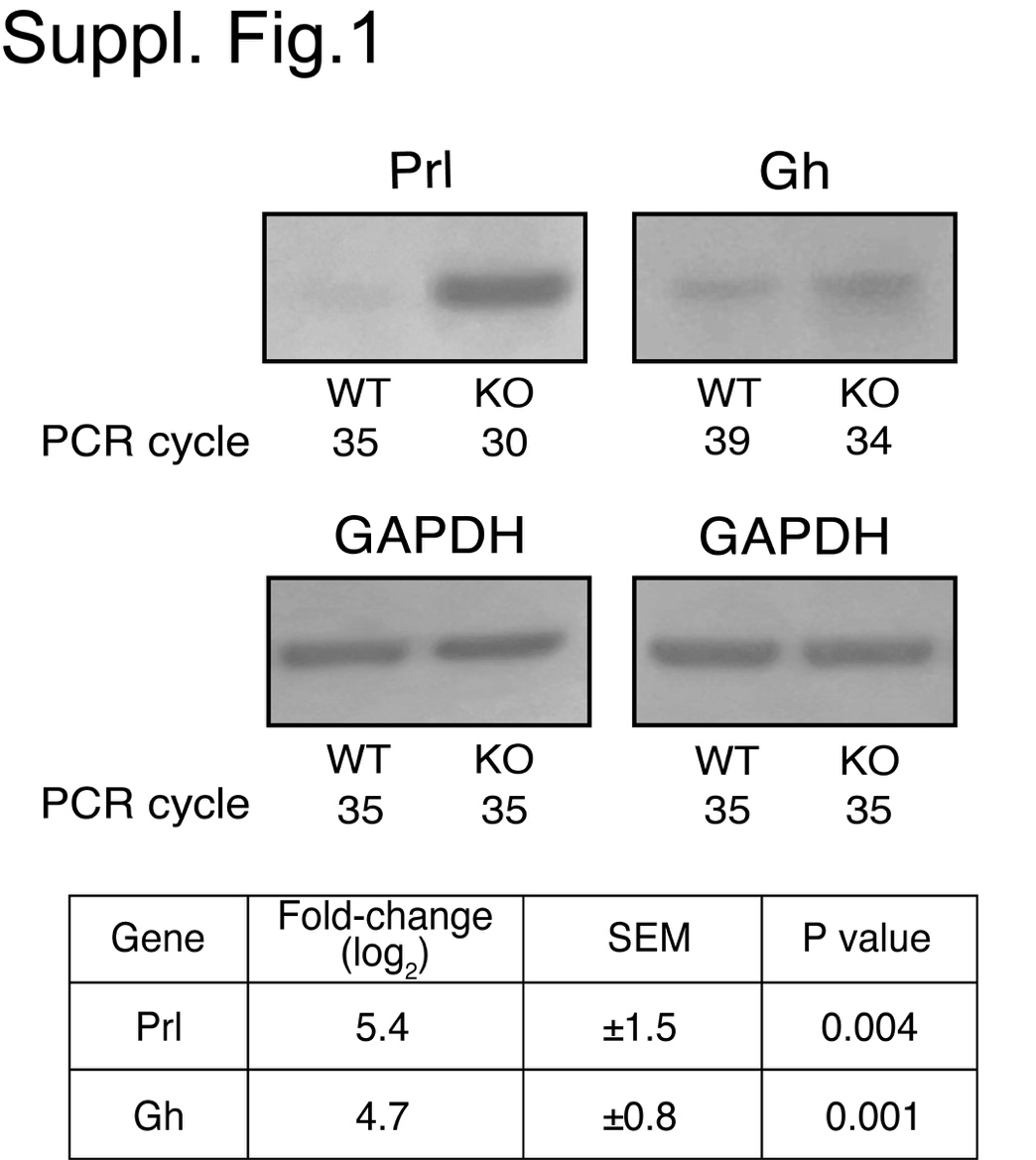
**

**Suppl. Fig. 1. Prl and Gh mRNA expression in the cerebral cortexes of control and DGKη-KO mice as measured by RT-PCR**

*Gh* and *Prl* mRNA expression in the cerebral cortexes of 12-week-old male control and DGKδ-KO mice were measured by RT-PCR using different cycles as indicated. The amplified bands of *Gh* and *Prl* were scanned and quantified using the ImageJ software, then normalized relative to the GAPDH levels. The band intensities in the control mouse were set to 1 (log_2_1 = 0) and the expression of *Gh* and *Prl* as being altered was determined. Data are shown as the means ± SEM of three animals per genotype (WT: n=3, KO: n=3).

**Suppl. Table 1. Identification of the acyl species in each PA molecular species in the mouse cerebral cortex**

| PA molecular species | Identified acyl chains | | | | | |
| --- | --- | --- | --- | --- | --- | --- |
| 28:0 | 10:0/18:0 (96.6%) | 12:0/16:0 (2.0%) | 14:0/14:0 (1.4%) |  |  |  |
| 30:2 | 10:0/20:2 (10.0%) | 12:0/18:2 (37.1%) | 14:1/16:1 (52.8%) |  |  |  |
| 30:1 | 10:0/20:1 (1.7%) | 12:0/18:1 (1.3%) | 14:0/16:1 (95.5%) | 14:1/16:0 (1.5%) |  |  |
| 30:0 | 10:0/20:0 (1.3%) | 12:0/18:0 (1.6%) | 14:0/16:0 (97.0%) |  |  |  |
| 32:3 | 14:1/18:2 (13.7%) | 14:0/18:3 (86.3%) |  |  |  |  |
| 32:2 | 10:0/22:2 (1.6%) | 14:0/18:2 (21.0%) | 14:1/18:1 (0.1%) | 16:1/16:1 (77.2%) |  |  |
| 32:1 | 10:0/22:1 (0.7%) | 14:0/18:1 (4.4%) | 16:0/16:1 (94.9%) |  |  |  |
| 32:0 | 10:0/22:0 (0.6%) | 14:0/18:0 (7.4%) | 16:0/16:0 (92.0%) |  |  |  |
| 34:5 | 14:0/20:5 (96.1%) | 14:1/20:4 (3.9%) |  |  |  |  |
| 34:4 | 14:0/20:4 (58.0%) | 16:1/18:3 (42.0%) |  |  |  |  |
| 34:3 | 14:0/20:3 (3.2%) | 16:0/18:3 (57.1%) | 16:1/18:2 (39.7%) |  |  |  |
| 34:2 | 16:0/18:2 (47.3%) | 16:1/18:1 (52.7%) |  |  |  |  |
| 34:1 | 10:0/24:1 (0.8%) | 14:0/20:1 (0.1%) | 16:0/18:1 (97.2%) | 16:1/18:0 (1.9%) |  |  |
| 34:0 | 10:0/24:0 (1.2%) | 14:0/20:0 (0.4%) | 16:0/18:0 (98.4%) |  |  |  |
| 36:6 | 14:0/22:6 (11.9%) | 16:1/20:5 (87.6%) | 18:3/18:3 (0.5%) |  |  |  |
| 36:5 | 14:0/22:5 (0.1%) | 16:0/20:5 (97.0%) | 16:1/20:4 (2.3%) | 18:2/18:3 (0.6%) |  |  |
| 36:4 | 14:0/22:4 (0.1%) | 16:0/20:4 (89.2%) | 16:1/20:3 (0.4%) | 18:1/18:3 (6.0%) | 18:2/18:2 (4.3%) |  |
| 36:3 | 16:0/20:3 (4.8%) | 16:1/20:2 (0.9%) | 18:0/18:3 (9.9%) | 18:1/18:2 (84.5%) |  |  |
| 36:2 | 14:0/22:2 (0.1%) | 16:0/20:2 (4.7%) | 16:1/20:1 (0.4%) | 18:0/18:2 (6.7%) | 18:1/18:1 (88.2%) |  |
| 36:1 | 14:0/22:1 (0.1%) | 16:0/20:1 (4%) | 16:1/20:0 (0.1%) | 18:0/18:1 (95.8%) |  |  |
| 36:0 | 10:0/26:0 (1.3%) | 14:0/22:0 (0.1%) | 16:0/20:0 (13.4%) | 18:0/18:0 (85.2%) |  |  |
| 38:6 | 16:0/22:6 (44.1%) | 16:1/22:5 (0.4%) | 18:1/20:5 (53.9%) | 18:2/20:4 (1.6%) |  |  |
| 38:5 | 16:0/22:5 (6.2%) | 16:1/22:4 (0.1%) | 18:0/20:5 (76.1%) | 18:1/20:4 (17.5%) | 18:2/20:3 (0.1%) |  |
| 38:4 | 16:0/22:4 (5.9%) | 18:0/20:4 (91.7%) | 18:1/20:3 (2.1%) | 18:2/20:2 (0.3%) |  |  |
| 38:3 | 16:1/22:2 (1.1%) | 18:0/20:3 (38.8%) | 18:1/20:2 (57.6%) | 18:2/20:1 (2.5%) |  |  |
| 38:2 | 16:0/22:2 (5.4%) | 16:1/22:1 (2.8%) | 18:1/20:1 (90.7%) | 18:2/20:0 (1%) |  |  |
| 38:1 | 14:0/24:1 (0.1%) | 16:0/22:1 (6.1%) | 16:1/22:0 (1.8%) | 18:0/20:1 (72%) | 18:1/20:0 (20%) |  |
| 38:0 | 14:0/24:0 (1.1%) | 16:0/22:0 (56.9%) | 18:0/20:0 (42%) |  |  |  |
| 40:6 | 18:0/22:6 (75.1%) | 18:1/22:5 (11.5%) | 18:2/22:4 (0.2%) | 20:1/20:5 (11.8%) | 20:2/20:4 (0.8%) | 20:3/20:3 (0.7%) |
| 40:5 | 18:0/22:5 (65.1%) | 18:1/22:4 (13%) | 20:0/20:5 (6.2%) | 20:1/20:4 (15.5%) | 20:2/20:3 (0.2%) |  |
| 40:4 | 18:0/22:4 (17.4%) | 18:2/22:2 (3.5%) | 18:3/22:1 (0.1%) | 20:0/20:4 (54.7%) | 20:1/20:3 (21.4%) | 20:2/20:2 (2.9%) |
| 40:3 | 18:1/22:2 (57.8%) | 18:2/22:1 (4.7%) | 20:0/20:3 (6.1%) | 20:1/20:2 (31.4%) |  |  |
| 40:2 | 16:1/24:1 (4.9%) | 18:0/22:2 (8.5%) | 18:1/22:1 (80.2%) | 18:2/22:0 (1.5%) | 20:0/20:2 (4.8%) |  |
| 40:1 | 16:0/24:1 (60.3%) | 16:1/24:0 (6.3%) | 18:0/22:1 (3.2%) | 18:1/22:0 (28.5%) | 20:0/20:1 (1.8%) |  |
| 40:0 | 16:0/24:0 (94.1%) | 18:0/22:0 (5.7%) | 20:0/20:0 (0.2%) |  |  |  |

^a^ The relative abundance (%) was based on the peak areas of the fragment ions (ESI-MS/MS) for each molecular ion.
